# Analysis of ALS-related proteins during herpes simplex virus-2 latent infection

**DOI:** 10.1186/s12974-020-02044-4

**Published:** 2020-12-07

**Authors:** Jorge Rubén Cabrera, Ignacio Rodríguez-Izquierdo, José Luis Jiménez, María Ángeles Muñoz-Fernández

**Affiliations:** 1 Sección Inmunología, Laboratorio InmunoBiología Molecular, Hospital General Universitario Gregorio Marañón, Madrid, Spain; 2 Instituto de Investigación Sanitaria Gregorio Marañón (IiSGM), Madrid, España; 3 Plataforma de Laboratorio, Hospital General Universitario Gregorio Marañón, Madrid, Spain; 4 Spain HIV HGM BioBanco, Madrid, Spain; 5 Networking Research Center on Bioengineering, Biomaterials and Nanomedicine (CIBER-BBN), C/Dr. Esquerdo 46, 28007 Madrid, Spain

**Keywords:** Herpes simplex virus, Latency, Spinal cord, Neurodegeneration, ALS, Microglia, C9ORF72

## Abstract

**Background:**

Genetics have provided hints on potential molecular pathways involved in neurodegenerative diseases (NDD). However, the number of cases caused exclusively by genetic alterations is low, suggesting an important contribution of environmental factors to NDDs. Among these factors, viruses like herpes simplex viruses (HSV-2), capable of establishing lifelong infections within the nervous system (NS), are being proposed to have a role in NDDs. Despite promising data, there is a significant lack of knowledge on this and an urgent need for more research.

**Methods:**

We have set up a mouse model to study HSV latency and its associated neuroinflammation in the spinal cord. The goal of this model was to observe neuroinflammatory changes caused by HSV latent infections, and if those changes were similar to alterations observed in the spinal cord of amyotrophic lateral sclerosis (ALS) patients.

**Results:**

In infected spinal cords, we have observed a strong leukocyte infiltration and a severe alteration of microglia close to motor neurons. We have also analyzed ALS-related proteins: we have not found changes in TDP-43 and Fus in neurons, but interestingly, we have found decreased protein levels of C9orf72, of which coding gene is severely altered in some familial forms of ALS and is critical for microglia homeostasis.

**Conclusions:**

Latent infection of HSV in the spinal cord showed altered microglia and leukocyte infiltration. These inflammatory features resembled to those observed in the spinal cord of ALS patients. No changes mimicking ALS neuropathology, such as TDP-43 cytoplasmic inclusions, were found in infected spinal cords, but a decrease in protein levels of C9orf72 was observed. Then, further studies should be required to determine whether HSV-2 has a role in ALS.

## Background

Neurodegenerative diseases (NDDs) are devastating conditions caused by a degeneration and death of neurons, affecting cognition, behavior, and motor functions. In a limited number of cases, the unique cause of the NDD is a genetic alteration, however, the majority of NDDs cases are idiopathic [1, 2]. This suggests that environmental factors may play an important role in NDDs, either being a direct cause of neurodegeneration or being risk factors for neurodegeneration.

Neurotropic viruses have been proposed to be environmental factors having an impact on NDDs [3, 4]. Among these, herpes simplex viruses (HSV) are the most efficient reaching and establishing long-term infections within the nervous system (NS) [5]. Herpes simplex virus types 1 and 2 (HSV-1 and HSV-2) are members of the herpes virus family of DNA viruses. They are extremely efficient human pathogens with symptoms that range from asymptomatic infections and cold/genital sores to severe neurological diseases when replicate within the NS [6, 7]. HSV initially infect skin or mucosal epithelia, where viruses start to replicate. Then, HSV gain access to the sensory free nerve endings innervating the infected epithelium. Once the nerve ending is infected, HSV particles are retrogradely transported to the cell body of the neuron. When HSV reach the neural cell body, viral DNA is released into the nucleus. During this process, neuronal antiviral response alerts the immune system (IS) and leukocytes get infiltrated into the NS [8, 9]. After that, two different outcomes may occur: if the IS and the neuronal antiviral responses are unable to control HSV lytic gene expression program, viruses replicate and infect neighbor and higher-order neurons. However, if the lytic infection is contained, then, HSV latency-associated transcripts (LATs) and several viral microRNAs are expressed silencing lytic gene expression program and allowing the establishment of latency [5, 10]. Latency is a major evolutionary advantage for HSV, as it makes these viruses escape from the IS and remain in the longest living cells in the body. However, latency is not a stationary state, and evidence suggest HSV DNA remains active during latency [11–13], forcing the IS to surveille constantly infected neurons, maintaining a persistent neuroinflammation [8, 9].

HSV capability to establish lifelong infections in the NS and the persistent neuroinflammation associated to latency make HSV potential candidates to have a role in NDDs [3, 14]. This subject is a matter of great controversy, although recent results are shedding light upon this unsolved question [14–18]. These studies are focused on HSV-1 and Alzheimer’s disease, as HSV-1 is the major cause of cold sores and the virus may gain access to the brain through the trigeminothalamic tracts. However, little attention has been paid to HSV-2, which is the major cause of genital herpes [19] and its capability to gain access to the spinal cord (SC) [20], although some reports have already related HSV to the most common of the lower motor neuron diseases, amyotrophic lateral sclerosis (ALS) [21–23].

ALS is one of the most aggressive NDDs, with a life expectancy of 3 to 5 years once is diagnosed. During the course of ALS, motor functions decline fast, speech is progressively lost, and finally, control of respiratory muscles fails, causing death [24, 25]. About 70% of ALS cases have a “limb-onset,” suggesting that motor neurons in the SC are initially affected. Five to ten percent of ALS cases have a family history of ALS or frontotemporal dementia (FTD), known as familial ALS, (fALS), whereas the rest of the cases are sporadic (sALS). ALS has distinctive neuropathological hallmarks; in approximately 95% of cases, TDP-43, a nuclear RNA-binding protein (RBP), appears to form cytoplasmic aggregates in motor neurons, whereas juvenile forms of ALS show aggregates of the RBP Fus [26]. Also, an important immune alteration is observed in the SC of ALS patients with microglial activation and infiltration of macrophages [27–31]. The cause of ALS remains unknown, although a great effort has been done to find the molecular causes of ALS. Viruses have been proposed to be an environmental factor for ALS [32], such as HSV [21–23], however, we lack of evidence to confirm this association. Curiously enough, several genes associated with ALS [33], such as *SQSTM1/P62* or *TBK1*, are involved in autophagy and innate immune response, two critical defense mechanisms used by neurons and glial cells to control HSV infections [34–36].

One of those genes involved in autophagy and innate immune response altered in some ALS patients is *Chromosome 9 Open Reading Frame 72* (*C9ORF72*). Mutations in *C9ORF72* are the most frequent cause of fALS in Europe and North America [33, 37, 38]. *C9ORF72* codes for a guanine nucleotide exchange factor (GEF) protein involved in a wide range of vesicular trafficking events reviewed in [39, 40], including the regulation of presynaptic vesicles [41] and the control of the lysosome and the latest steps of autophagy [42]. *C9ORF72* gene has a hexanucleotide repeated region (GGGGCC) located between exons 1a and 1b. Healthy individuals have usually ≤ 11 hexanucleotide repeats. When the number of hexanucleotide repeats is higher than 30, it is considered to be pathogenic [39, 40]. Three potential mechanisms have been proposed for *C9ORF72* mutation toxicity, two gain-of-function mechanisms related to RNA toxicity and aberrant RNA translation, and a loss-of-function mechanism [39, 40]. Different experimental mouse models have shown that loss-of-function of *3110043O21Rik* gene, the mouse ortholog of *C9ORF72* (referred from here as *C9orf72*), causes a severe immune dysregulation including lymphadenopathy, splenomegaly, and autoimmune disease [43–45]. This is partially caused by a severe alteration of macrophages and microglia [43, 46]. Curiously, these immune cells are activated and required for the control of HSV infections within the central nervous system (CNS) [9, 47–49].

All these evidences have encouraged us to establish an HSV latent infection mouse model in the SC, to test whether changes caused by HSV latent infection and its associated neuroinflammation. Then, we have studied whether those changes could show similarities to alterations observed in the SC of ALS patients. We have used HSV-2 genital infection as a natural route to get access to the SC. We have characterized changes in the SC of infected mice by histochemistry (HC), immunofluorescence (IF), and Western blot (WB). At the cellular level, we have observed a severe leukocyte infiltration and microglial alteration in infected SCs. At the molecular level, we have not observed changes in TDP-43 and Fus in neurons, but we have observed a decrease in the ALS-related protein C9orf72. These observations suggest that HSV-2 latent infection is not sufficient to induce ALS neuropathological hallmarks. However, neuroinflammatory features observed close to motor neuron will require further studies to evaluate whether HSV may be a risk factor for ALS.

## Methods

### Virus, mice, HSV-2 vaginal infection, and in vivo characterization of the model

The HSV-2 strain 333 (GenBank accession number LS480640) was propagated and plaqued on Vero cells (ATCC CCL-81, Manassas, VA, USA), using standard practices, as previously described [50]. Stock of HSV-2 strain 333 was prepared and titrated by plaque assay and stored at −80 °C.

Seven-week-old (w.o.) female mice Balb/cByJ were purchased from Charles Rivers. They were maintained for 1 week in the animal facility before any experimental manipulation. Prior to vaginal HSV-2 challenge, mice received a 2 mg subcutaneous injection of medroxyprogesterone acetate (Depo-Provera (Depo), Pfizer) to increase susceptibility to HSV-2 infection. Four days later, mice were anesthetized with isoflurane (2-chloro-2-(difluoromethoxy)-1,1,1-trifluoro-ethane; Forane, Abbott)), inoculated intravaginally with HSV-2 (50.000 PFU in 10 μL diluted in serum free DMEM) or with Vero cell extract diluted in serum free DMEM (Mock) and then maintained in a supine position for 15 min post-infection. Infection day was considered as 0 day post-infection (DPI). Acyclovir (250 mg powder, Altan), was added to water drinking bottles at 1.3 mg/mL on 4 DPI, replaced every week, and maintained for 2 weeks until 18 DPI.

Mice were examined every 2 days for body weight and genital pathology. Disease score was graded according to a 4-point scale as previously reported [50]: 0, no apparent infection; 1, genital erythema; 2, moderate genital infection; 3, purulent genital ulceration with hair loss; and 4, severe ulceration of genital and surrounding tissue, hind limb paralysis (leading to euthanasia).

### SC dissection

Twenty-four DPI mice were euthanized by CO_2_ overdose and decapitated. SCs were dissected by hydraulic extrusion with PBS and cut in half at the beginning of the lumbar enlargement. Cervical and thoracic parts of the SC were ruled out whereas the lumbo-sacral part of the SC was stored at −80 °C when used for qPCR and WB or fixed in 4% paraformaldehyde in PBS for 6 h when used for HC or IF.

### RNA isolation and real-time qPCR

RNA was isolated using RNeasy Mini Kit from Qiagen according to the manufacturer’s instructions. cDNA was synthesized using the Promega GoScript™ Reverse Transcription System (Thermo Fisher Scientific) using random primers. Real-time quantitative PCR (RT-qPCR) was performed using FastStart Universal SYBR Green Master (Rox) (Roche Merck) adjusted to a final volume of 15 μL. RT-qPCR was performed in a Stratagene Mx3005P qPCR system and results were analyzed using the MxPro software.

Oligonucleotides were as follows:

- LAT F 5′ GTC AAC ACG GAC ACA CTC TTT T 3′
- LAT R 5′ CGA GGC CTG TTG GTC TTT ATC 3′
- Mouse GAPDH F 5′ AGG TCG GTG TGA ACG GAT TTG 3′
- Mouse GAPDH R 5′ TGT AGA CCA TGT AGT TGA GGT CA 3′

### Vibratome sectioning

Once fixed, SCs were washed three times in PBS for 10 min, then embedded in prewarmed saccharose-agarose (10-4%) and left to settle overnight. On the next day, SCs were recovered from the agarose-saccharose block and sectioned horizontally in a Campden Instruments 7000MZ-2 vibratome (40 μm sections). Sections were stored in a cryoprotectant storage solution (ethylene glycol 300 mL, glycerol 300 mL, PBS 10X 100 mL, distilled water 300 mL) and kept at −20 °C until they were used.

### Nissl staining

Three to six random sections from different regions were used per SC analyzed. Sections were washed twice in PBS and then permeabilized using a 50% alcohol/water solution for 30 min. Then, sections were washed in deionized distilled water for 3 min and submerged in an activated Cresyl Violet (Merck) filtered solution for 30 min. Sections, then, were washed in distilled water for 3 min and decolored in 70% alcohol/water for a minimum time of 30 min. When white matter was decolored, sections were submerged three times in 100% alcohol for 5 min and then in isoparaffin H twice for 5 min. Finally, sections were allowed to dry for 3 min and mounted in DePeX mounting medium (Serva).

### Immunofluorescence

Six random sections from different regions were used per SC analyzed. Sections were washed twice in PBS and then Tris-EDTA antigen retrieval was performed (30 min, 65 °C). Sections were washed in PBS and permeabilized/blocked using PBS 1% Triton X-100 3% BSA solution for 1 h. Primary antibodies were incubated in PBS 1% Triton X-100 3% BSA for 2 h at room temperature and then overnight at 4 °C. The next day, sections were washed three times in PBS for 10 min and then incubated with secondary antibodies in PBS 1% Triton X-100 3% BSA for 2 h at room temperature. Finally, sections were washed in PBS, PBS-Dapi, and PBS for 10 min and mounted using Fluorescence Mounting Medium (Agilent Dako).

#### Primary antibodies

Rat anti CD45 Clone 30-F11 (used 1/200) and rat anti Lamp1Clone 1D4B (used 1/20) were from Biolegend. Rat anti-Mouse I-A/I-E (MHC II) Clone 2G9 (used 1/100) was from BD Pharmigen. Chicken anti-NeuN ABN91 (used 1/1000) was from Millipore. Rabbit anti Iba1 019-19741 (used 1/500) was from Wako. Rabbit anti TDP-43 10782-2-AP (used 1/500), rabbit anti-Fus 11570-1-AP (used 1/500), and rabbit anti-C9ORF72 22637-1-AP (used 1/500) were from Proteintech.

#### Secondary antibodies

Goat anti-Rat IgG Alexa Fluor 488, Donkey anti-Rabbit IgG Alexa Fluor 555, and Goat anti-Chicken IgY Alexa Fluor 647 were from Thermo Fisher Scientific.

Sections were analyzed in a Leica TCS SPE confocal microscope and pictures were analyzed using Leica Application Suite X and quantified using the NIH Fiji software.

### Western blot

Extracts were prepared by homogenizing SCs using an ice-cold glass tissue homogenizer in ice-cold RIPA-like extraction buffer composed by Tris-HCl pH 7.4 (50 mM), NaCl (150 mM), Triton X-100 (1%), sodium deoxylcholate (0.5%), SDS (0.1%), NaF (5 mM) plus protease inhibitor complete, Mini (Roche), and phosphatase inhibitor PhosStop (Roche), both used according to manufacturer instructions. Samples were homogenized and centrifuged at 15,000×*g* for 15 min at 4 °C. Resulting supernatants were collected and protein content was determined by Bradford assay. Fifteen to twenty micrograms of total protein were electrophoresed on 8, 10, or 12% SDS-polyacrylamide gel and transferred to a PVDF membrane and blocked in TBS-T (150 mM NaCl, 20 mM Tris–HCl, pH 7.5, 0.05% Tween 20) with 3% BSA.

#### Primary antibodies

Rabbit anti β-actin Poly6221 (used 1/1000) and rat anti P2ry12 clone S16007D (used 1/500) were from Biolegend. Rabbit anti Iba1 019-19741 (used 1/1000) was from Wako. Rabbit anti-TDP-43 10782-2-AP (used 1/2000), rabbit anti-Fus 11570-1-AP (used 1/1000), rabbit anti-C9ORF72 22637-1-AP (used 1/2000), and rabbit anti-SOD1 10269-1-AP (used 1/1000) were from Proteintech.

#### Secondary antibodies

Anti-rat IgG HRP-linked #7077S and Anti-rabbit IgG HRP-linked #7074S were from cell signaling.

Membranes were submerged in Clarity™ Western ECL Substrate (Bio-Rad) and pictures were taken in an UVItec Alliance 4.7 and quantified using the NIH Fiji software.

## Results

**Fig. 1:**
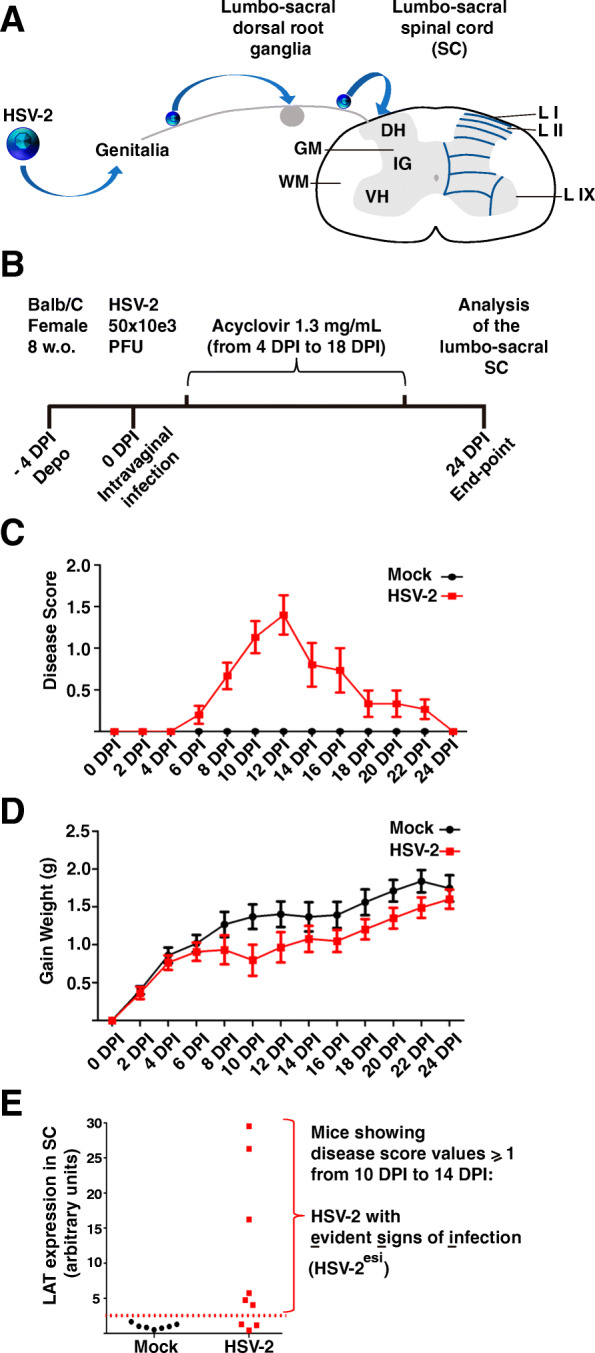
Mouse model of HSV-2 latent infection in the SC. **a** Anatomical scheme explaining HSV-2 access into the SC through a genital infection and simplified scheme of the SC: WM (white matter), GM (gray matter), DH (dorsal horn), IG (intermediate gray), VH (ventral horn), L I (lamina I), L II (lamina II), L IX (lamina IX) **b** Scheme of the HSV-2 latently infected SC mouse model. **c** Disease score of Mock and HSV-2 infected mice; measurements were performed every 2 days (*n* = 15). **d** Gain weight of Mock and HSV-2 infected mice; measurements were performed every 2 days (*n* = 15). **e** q-PCR analysis of LAT expression in the SC of Mock (*n* = 7) and HSV-2 (*n* = 10) from two independent experiments. An arbitrary cut-off value 3 (dotted-line) was established to consider a SC positive for LAT expression. Disease score values and LAT expression defined the HSV-2^esi^ category used in this manuscript

HSV-2 generally infects genital skin and mucosa and gets access into lumbo-sacral dorsal root ganglia innervating genital organs. In some patients, HSV-2 can spread into the lumbo-sacral area of the SC causing severe complications when the virus replicates there [20]; however, there are few data about how often HSV-2 reaches the SC in subclinical and genital infections. Access of HSV-2 to the SC through sensory neurons (Fig. 1a) is supported by mouse models [21, 51], although access through the parasympathetic innervation of genital organs has been also proposed [52]. In order to study long-term effects of HSV latency and its associated neuroinflammation in the SC, we have established an HSV-2 latent infection in mice (Fig. 1b and the “Methods” section). In brief, mice were Mock or HSV-2 (50,000 PFU) vaginally infected. Four days later, when HSV-2 reaches the SC [21, 51], we started to treat mice with oral acyclovir; the usage of this antiviral drug inhibits HSV-2 replication, force the virus to activate the latency program, and prevents death of infected mice. Treatment was maintained for 2 weeks, until 18 DPI. Twenty-four DPI, more than 95% of infected mice survived and we considered that HSV-2 has established latency. Signs of vaginal pathology were monitored every 2 days (Fig. 1c and the “Methods” section). Infected mice showed signs of vaginal inflammation by 6DPI, signs peaked at 12DPI, being the average score around 1.5 (Fig. 1c and the “Methods” section). Then, mice were recovering until no signs of vaginal inflammation were detectable by 24 DPI. We also monitored gain weight during infection (Fig. 1d). Infected mice loss weight between 8 and 10 DPI; however, by 24 DPI, gain weight was similar between Mock and HSV-2 infected mice. Finally, we tested the establishment of HSV-2 latency in the lumbo-sacral area of the SC. We stained SC sections using HSV antibodies; usually, no viral antigens were detected in infected SCs (not shown), although in rare occasions isolated cells appear to be positive for HSV, a phenomenon expected during latency [11, 12]. Finally, to assure that latency was established in the lumbo-sacral area of the SC, we performed q-PCR to detect levels of HSV-2 LAT (Fig. 1e). Background signal in Mock SCs was arbitrarily considered as value 1. In HSV-2 infected SCs, we found two different populations: one showing similar values than Mock (~ 30% of SCs) that was considered as LAT^−^, and another population showing LAT values higher than 3 (~ 70% of SCs) considered positive for LAT expression (Fig. 1e). Then, we combined LAT expression data with disease score values, and this allowed us to define a group of mice denominated HSV-2 with evident sign of infection in mice (HSV-2^esi^). HSV-2^esi^ were those mice with sustained signs of vaginal inflammation (score 1 or higher) at 10, 12, and 14 DPI. All mice considered as HSV-2^esi^ showed LAT values in the SC higher than 4 (Fig. 1e). Thereby, we only used Mock and HSV-2^esi^ mice for the rest of this study, as only in HSV-2^esi^ mice, we can assure that HSV-2 has reached the SC and latency was correctly established there.

**Fig. 2:**
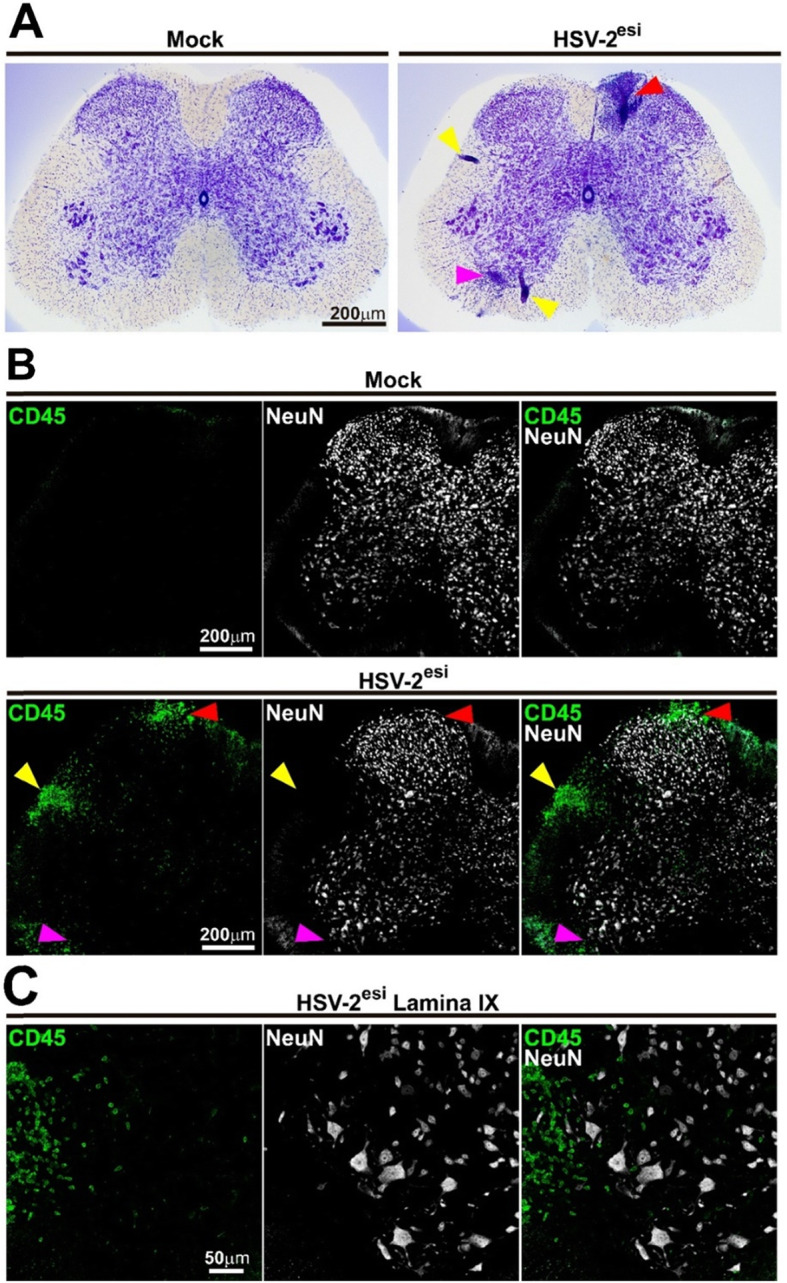
Leukocyte infiltration in the SC of HSV-2^esi^ latently infected mice. **a** Nissl staining of Mock (*n* = 4) and HSV-2^esi^ (*n* = 5) SCs from two independent experiments. Red arrowhead points to a cell accumulation spanning across laminae I and II in the dorsal horn and the gracile fasciculus, yellow arrowhead points to cell accumulations within the white matter, and magenta arrowhead points to a cell accumulation in lamina IX in the ventral horn. **b** Single-plane confocal images of Mock (*n* = 4) and HSV-2^esi^ (*n* = 5) SCs from two independent experiments. CD45 in green and NeuN in white. Red arrowhead points to a CD45^high^ cell accumulation in lamina I in the dorsal horn, yellow arrowhead points to a CD45^high^ cell accumulation within the white matter, and magenta arrowhead points to a CD45^high^ cell accumulation in the ventral horn. **c** Z-stack confocal images of an HSV-2^esi^ SC (lamina IX). CD45 in green and NeuN in white

Once the HSV-2^esi^ category was established, we performed Nissl staining in order to analyze the morphology of infected SCs (Fig. 2a). HSV-2^esi^ SCs showed a similar structure to Mock SCs, and no significative loss of neurons was observed. Major difference observed in HSV-2^esi^ SCs was the abnormal and massive appearance of small and deeply stained cells (Fig. 2a, arrowheads), especially in lumbar 6, sacral 1, and sacral 2 regions. All HSV-2^esi^ SCs analyzed showed the presence of these clusters of cells in the dorsal horn, spanning across laminae I and II, and also, invading the gracile fasciculus (Fig. 2a, red arrowhead). We also found frequent accumulation of these cells in discrete areas of the white matter (Fig. 2a, yellow arrowheads). Finally, frequently, we observe the accumulation of these cells in the ventral horn, within lamina IX (Fig. 2a, magenta arrowhead). We suspected these cells could be infiltrating leukocytes, in agreement to observations made in the SC of HSV-2 genitally infected guinea pigs [53]. To confirm that, we stained SC sections with the leukocyte marker anti-CD45 and the neuronal marker anti-NeuN (Fig. 2b). Mock sections showed few and scattered CD45^high^ cells in the meningeal area, whereas we could confirm that cell accumulations observed in the dorsal horn (Fig. 2b, red arrowhead), white matter (Fig. 2b, yellow arrowhead), and ventral horn of HSV-2^esi^ mice (Fig. 2b, magenta arrowhead) were clusters of CD45^high^ cells. We focused on the presence of CD45^high^ cells in the ventral horn of infected SC (Fig. 2c). In general, CD45^high^ cells there appeared in clusters within the white matter, but still, several CD45^high^ cells were found in close contact to large neurons in the lamina IX, identified by morphology, size, and location as motor neurons. These results showed infiltration of leukocytes in the SC during HSV-2 latency. As expected, this infiltration of CD45^high^ cells was higher in the dorsal horn, the area of the SC that receives the sensory input from genital organs. Also, CD45^high^ cells were found in the intermediate gray of the sacral region, in several spots within the white matter, and frequently, in close proximity to motor neurons.

**Fig. 3:**
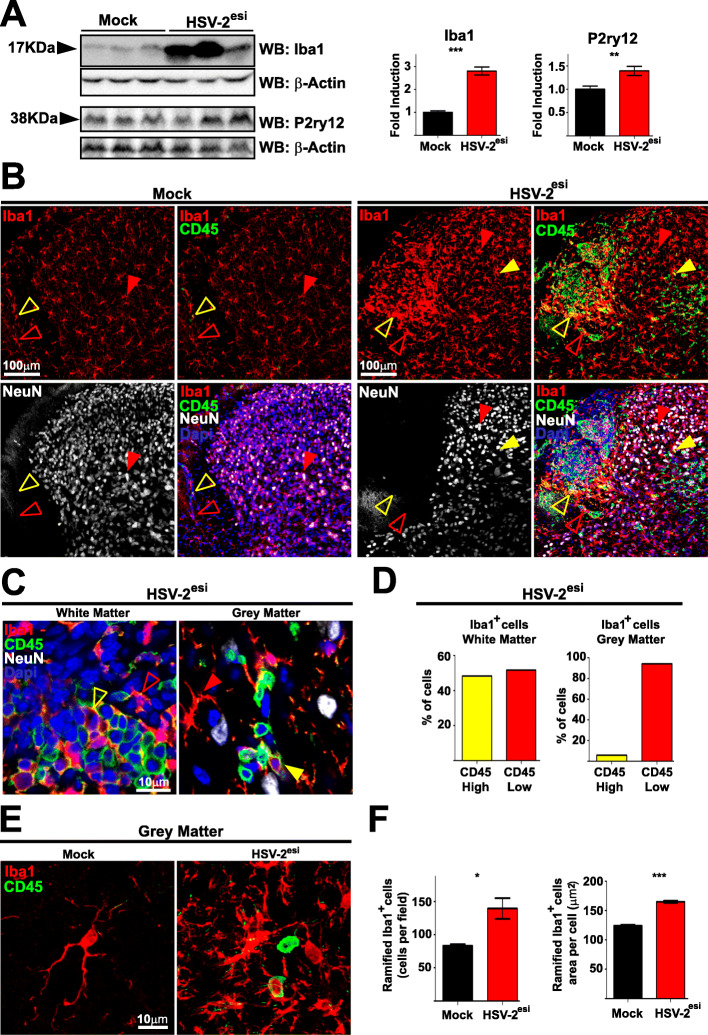
Microglia alteration in the SC of HSV-2^esi^ latently infected mice. **a** WBs for Iba1 (Mock and HSV-2^esi^ (*n* = 7 from two independent experiments)) and P2ry12 (Mock and HSV-2^esi^ (*n* = 8 from two independent experiments)). ***p < 0.001, **p < 0.01. Each lane corresponds to one SC. **b** Z-stack confocal images of Mock (*n* = 4) and HSV-2^esi^ (*n* = 5) SCs from two independent experiments. CD45 in green, Iba1 in red, NeuN in white, and Dapi in blue. Open red arrowheads point to microglia in the white matter; solid red arrowheads point to microglia in the gray matter; open yellow arrowheads point to macrophages (MΦ) in the white matter; solid red arrowheads point to MΦ in the gray matter. **c** Single-plane confocal images of Mock (*n* = 4) and HSV-2^esi^ (*n* = 5) SCs from two independent experiments. CD45 in green, Iba1 in red, NeuN in white, and Dapi in blue. Open red arrowheads point to microglia in the white matter; solid red arrowheads point to microglia in the gray matter; open yellow arrowheads point to MΦ in the white matter; and solid yellow arrowheads point to MΦ in the gray matter. **d** Quantification of CD45^high^ Iba1^+^ versus CD45^low^ Iba1^+^ cells in the white matter (left graph) and in the gray matter (right graph). *n* = 5 from two independent experiments. **e** Z-stack confocal images of Mock and HSV-2^esi^ SCs. CD45 in green, Iba1 in red. **f** Quantification of the number of ramified CD45^low^ Iba1^+^ cells in the gray matter. Mock (*n* = 4) and HSV-2^esi^ (*n* = 5) from two independent experiments (left graph). Quantification of the area of ramified CD45^low^ Iba1^+^ cells in the gray matter. Mock (*n* > 2000) and HSV-2^esi^(*n* > 3000) from two independent experiments (right graph). *p < 0.05, ***p < 0.001

Recent advances in genetics have pointed to innate immunity as a major player in NDDs [54, 55]. Microglia are the main innate immune cells within the CNS and they are critical to control HSV infections in the brain [9, 47–49]. Then, we decided to analyze the state of microglial cells in the SC of HSV-2^esi^ latently infected mice. Firstly, we measured levels of microglial markers by WB (Fig. 3a). Levels of Iba1 were elevated in HSV-2^esi^ SCs compared to Mock (Fig. 3a, upper panels). However, Iba1 is a marker of microglia, monocytes, and macrophages (MΦ). Therefore, we performed additional WBs analyzing the microglial specific marker P2ry12 (Fig. 3a, lower panels). Levels of P2ry12 were increased in HSV-2^esi^ SC compared to Mock, although to a lesser extent than Iba1. To further study changes occurring in microglia, we performed IFs using anti-Iba1 and anti-CD45 antibodies. Microglia was identified as Iba1^+^ cells with irregular ramified shapes and scattered staining of CD45, while MΦ appeared as rounded cells expressing high levels of CD45 [56]. In Mock sections, microglia were the main Iba1^+^ population found in the gray matter (Fig. 3b, left panels, solid red arrowhead), whereas in the white matter, we found microglia (Fig. 3b, left panels, open red arrowhead) and some MΦ (Fig. 3b, left panels, open yellow arrowhead). In HSV-2^esi^ sections, we could observe several changes. Microglia showed a more intense Iba1 staining, either in the gray or in the white matter (Fig. 3b, right panels, solid and open red arrowheads). Also, we observed an increase of MΦ, especially within the white matter (Fig. 3b, right panels, solid and open yellow arrowheads). We assured that CD45^high^ Iba1^+^ cells observed were not an artifact of section thickness and z stacking by performing higher magnification and single-plane imaging. This approach confirmed the presence of MΦ in the white matter and in the gray matter of HSV-2^esi^ SCs (Fig. 3c, solid and open yellow arrowheads). We quantified relative amounts of MΦ (CD45^high^ Iba1^+^) and microglia (CD45^low^ Iba1^+^) in HSV-2^esi^ SCs (Fig. 3d). Quantification revealed that presence of MΦ in the white matter was high, matching the number of microglia, while microglia was the predominant innate immune cell in the gray matter (Fig. 3d). Finally, we analyzed in detail changes in microglia (CD45^low^ Iba1^+^) in the gray matter (Fig. 3e and f). We found an increased number of microglia in HSV-2^esi^ SCs compared to Mock (Fig. 3f). Microglial morphology was also altered (Fig. 3e). Microglia in Mock sections showed the expected shape for homeostatic microglia with a small soma and long ramified processes [57]. In contrast, in HSV-2^esi^, microglial somas were bigger and the total area was increased, although cells still appeared ramified (Fig. 3f). Together, these results indicated that microglia were altered during HSV-2 latency in the SC, with increased number of microglial cells and having those higher areas. Also, we observed certain compartmentalization of innate immune cells during HSV-2 latency, as MΦ almost matched in number with microglia in the white matter, while microglia were the main innate immune population in the gray matter.

**Fig. 4:**
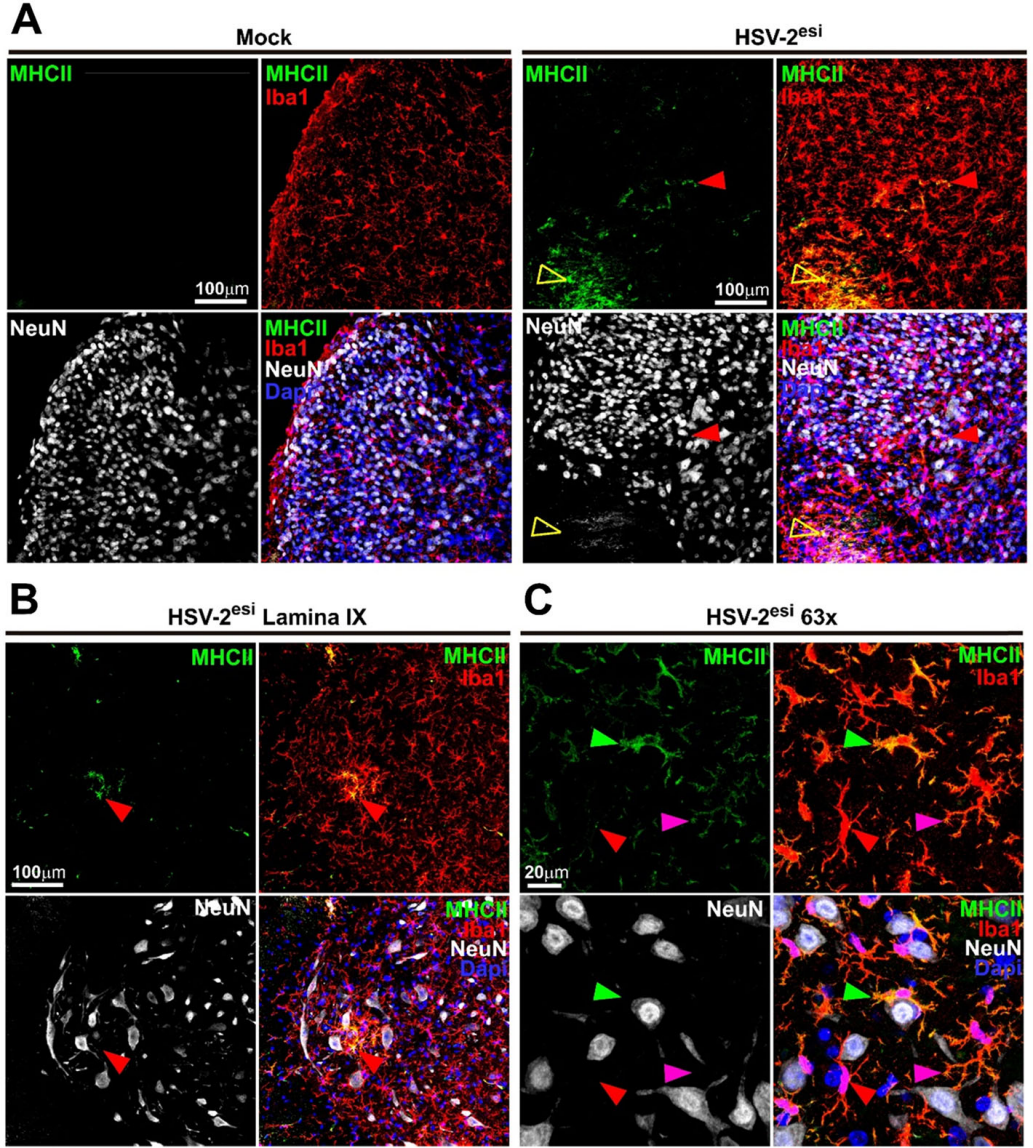
Differential expression of MHC-II in microglia during HSV-2^esi^ latent infection. **a** Z-stack confocal images of Mock (*n* = 4) and HSV-2^esi^ (*n* = 5) SCs from two independent experiments. MHC-II in green, Iba1 in red, NeuN in white, and Dapi in blue. Open yellow arrowhead point to a MHC-II^high^ cell in the white matter; solid red arrowhead point to a MHC-II^high^ cell in the gray matter. **b** Z-stack confocal images of HSV-2^es^ SCs on lamina IX. MHC-II in green, Iba1 in red, NeuN in white, and Dapi in blue. Red arrowhead points to a MHC-II^high^ cell close to motor neurons. **c** Z-stack confocal images of HSV-2^es^ SCs. MHC-II in green, Iba1 in red, NeuN in white, and Dapi in blue. Green arrowhead point to a high-expressing MHC-II cell, magenta arrowhead point to a moderate-expressing MHC-II cell, and red arrowhead point to a low-expressing MHC-II cell

We wanted to better understand changes in microglia during HSV-2 latency. We tested whether altered microglia in infected SCs had a pro-inflammatory or an anti-inflammatory state. HSV-2^esi^ microglia were negative for the pro-inflammatory marker CD86 (not shown) and we observed no change in the ant-inflammatory marker Arginase-1 (not shown). In agreement with these data, we did not find changes in levels of p-Stat1, p-Stat3, or p-Stat6 (not shown). Then, we focused our attention on MHC-II. In homeostatic microglia, MHC-II levels are low and it gets upregulated upon activation [58]. Importantly, in several HSV-1 encephalitis models, upregulation of microglial MHC-II was observed during latency [9, 48]. In Mock sections, MHC-II was almost undetectable (Fig. 4a, left panels). In HSV-2^esi^ infected sections, we found high number of MHC-II^high^ cells distributed irregularly across the sections. We found clusters of MHC-II^high^ Iba1^+^ cells in the white matter, compatible with the presence of activated MΦ (Fig. 4a, right panels, open yellow arrowhead). In the gray matter, we found disperse ramified MHC-II^high^ Iba1^+^ cells distributed randomly (Fig. 4a, right panels, solid red arrowhead). Curiously, these MHC-II^high^ Iba1^+^ “MHC-II hot-spots” were more frequent in the intermediate gray area and in the ventral horn, close to motor neurons (Fig. 4b, red arrowhead). We analyzed these MHC-II^high^ Iba1^+^ “MHC-II hot-spots” in the gray matter with higher magnification; we could confirm that MHC-II^high^ were indeed ramified Iba1^+^ cells, most likely microglia. Also, we observed different degrees of MHC-II expression, with high-expressing MHC-II microglia (Fig. 4c, green arrowhead), moderate-expressing MHC-II microglia (Fig. 4c magenta arrowhead) and low-expressing MHC-II microglia (Fig. 4c, red arrowhead). These results confirmed the presence of activated microglia in the SC of HSV-2^esi^ mice. Also, these results showed the existence of different immune active areas within the gray matter, with different degree of microglial activation.

**Fig. 5:**
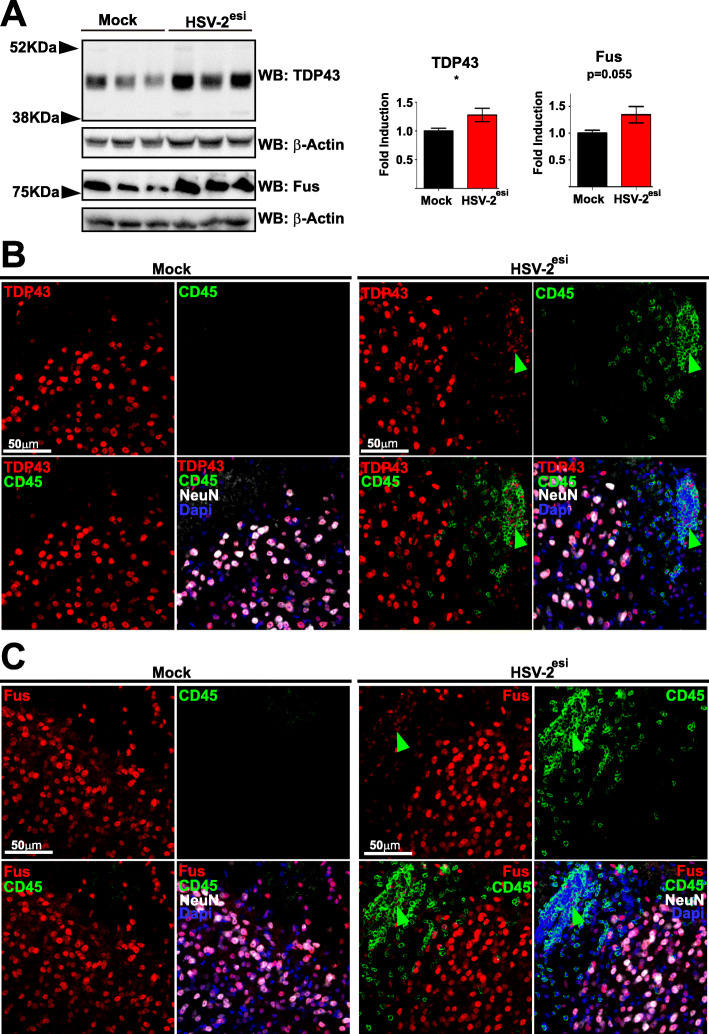
TDP-43 and Fus increased levels in the SC of HSV-2^esi^ latently infected mice. **a** WBs for TDP-43 (Mock and HSV-2^esi^ (*n* = 7 from two independent experiments)) and Fus (Mock and HSV-2^esi^ (*n* = 7 from two independent experiments)).*p < 0.05. Each lane corresponds to one SC. **b** Single-plane confocal images of Mock (*n* = 4) and HSV-2^esi^ (*n* = 5) SCs from two independent experiments. TDP-43 in red, CD45 in green, NeuN in white, and Dapi in blue. Red arrowhead points to a TDP-43^+^CD45^high^ cell. **c** Single-plane confocal images of Mock (*n* = 4) and HSV-2^esi^ (*n* = 5) SCs from two independent experiments. Fus in red, CD45 in green, NeuN in white, and Dapi in blue. Red arrowhead points to a Fus^+^CD45^high^ cell

Once HSV-2^esi^ microglia were characterized, we analyzed ALS-related proteins. We firstly focused on the nuclear RBP TDP-43. In more than 95% of cases of ALS, TDP-43 is hyperphosphorylated, degraded, and shows an aberrant cytoplasmic localization in motor neurons, independently whether they are familial or sporadic cases [59]. Firstly, we measured TDP-43 protein levels by WB. In HSV-2^esi^ SCs, levels of TDP-43 were increased compared to Mock (Fig. 5a, upper panels). We continued our analysis by measuring protein levels of Fus, another nuclear RBP altered in ALS, mainly in juvenile cases. Fus levels were also increased in HSV-2^esi^ SCs compared to Mock, although difference did not reach statistical significance (Fig. 5a, lower panels). In order to understand changes observed in TDP-43 and Fus, we performed IFs (Fig. 5b and c). As previously described, TDP-43 and Fus heavily stained the nucleus of neurons in the SCs (Fig. 5b and c). Regarding the infection state, we found no difference in the neuronal staining of TDP-43 and Fus, neither in the dorsal nor in the ventral horns. However, single-plane images revealed that several of the CD45^high^ infiltrated cells in HSV-2^esi^ were weakly, but indubitably, positive for TDP-43 (Fig. 5b, green arrowheads). The same was observed for Fus (Fig. 5c, green arrowhead). Altogether, these results suggested that TDP-43 and Fus had increased protein levels in HSV-2^esi^ SCs, although this increase was caused by leukocyte infiltration.

**Fig. 6:**
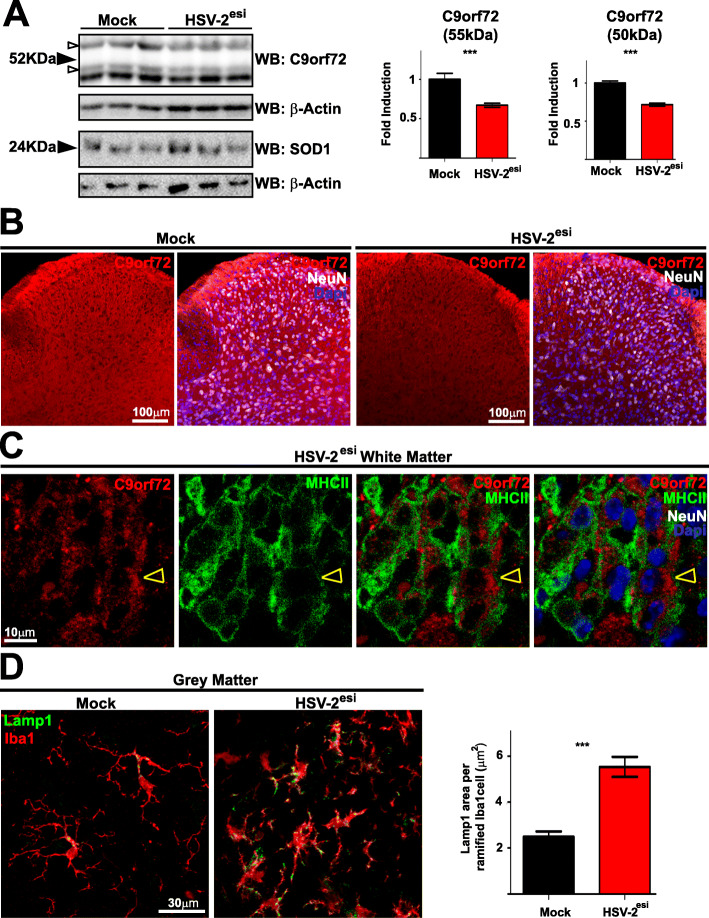
C9orf72 decreased levels in the SC of HSV-2^esi^ latently infected mice. **a** WBs for C9orf72 (Mock and HSV-2^esi^ (*n* = 8 from two independent experiments)) and SOD1 (Mock and HSV-2^esi^ (*n* = 7 from two independent experiments, *p* = 0.248)). Open arrowheads point to 55 kDa and 50 kDa C9orf72 isoforms. ***p < 0.001. Each lane corresponds to one SC. **b** Z-stack confocal images of Mock (*n* = 4) and HSV-2^esi^ (*n* = 5) SCs from two independent experiments. C9orf72 in red, NeuN in white, and Dapi in blue. **c** Single-plane confocal images of HSV-2^esi^SCs (*n* = 5) from two independent experiments. C9orf72 in red, MHC-II in green, NeuN in white, and Dapi in blue. Open yellow arrowhead points to a C9orf72^+^MHC-II^high^ cell in the white matter. **d** Z-stack confocal images of Mock (*n* = 3) and HSV-2^esi^ (*n* = 3) SCs. Lamp1 in green, Iba1 in red. Quantification of the area of Lamp1 in ramified Iba1^+^ cells in the gray matter. Mock and HSV-2^esi^ (*n* > 100) from three independent mice. ***p < 0.001

Then, we turned our attention to C9orf72. Mutations in the *C9ORF72* gene are the main cause of fALS and it causes around 5% of the sALS [37, 38]. *C9ORF72* is highly expressed in MΦ and microglia [43, 59]. In our model, we have observed an infiltration of MΦ and increased number of microglial cells in the HSV-2^esi^ SCs. These made us to hypothesize C9orf72 protein levels should be altered in infected SCs. WBs detected two C9orf72 isoforms in Mock SCs (Fig. 6a, upper panels, 55 and 50 kDa, open arrowheads). Interestingly, both isoforms of C9orf72 were decreased in HSV-2^esi^ SCs (Fig. 6a, upper panels, 55 and 50 kDa, open arrowheads). This result was confirmed using a different commercial antibody, although only one band (50 kDa) was observed (not shown). We wanted to know whether C9orf72 decrease was specific, or if this was observed in other fALS-related protein products. To do so, we analyzed protein levels of superoxide dismutase 1 (SOD1), as mutations in its coding gene are the second leading cause of fALS, and no change was found (Fig. 6a, lower panels). Then, we performed IFs using an anti-C9orf72. In Mock sections, C9orf72 antibody stained the SC parenchyma, (Fig. 6b, left panels) in agreement with the presynaptic and microglial functions of C9orf72 [41, 43]. C9orf72 staining in HSV-2^esi^ SCs showed a similar pattern than Mock SCs, but dimmer in signal across the entire section (Fig. 6b, right panels). This result was unexpected, then we decided to test whether C9orf72 was expressed in immune infiltrated cells, as was previously suggested. HSV-2^esi^ sections were stained using anti-C9orf72 and anti-MHC-II antibodies (Fig. 6c), and single-stack images revealed that MHC-II^high^ cells in the white matter were positive for C9orf72 (Fig. 6c, open yellow arrowheads). This extra load of C9orf72 carried by infiltrated immune cells suggested that changes in C9orf72 levels in HSV-2^esi^ SCs could be caused by changes in neurons and glial cells. To clarify that, we tried to co-stain C9orf72 with different presynaptic and microglial markers. However, due to technical issues, we could not perform that analysis (see the “Discussion” section). Then, as an indirect test for C9orf72 function in microglia, we analyzed the lysosome of ramified Iba1^+^. C9orf72 deficient mice show increased levels of Lamp1 in microglia and the same occurs in the SC of C9ORF72 ALS patients [43]. Therefore, if C9orf72 was decreased in microglia in HSV-2^esi^ SC, we should observe an increase in the levels of Lamp1 in those cells. As can be seen in Fig. 6d, levels of Lamp1 in ramified Iba1^+^ cells of HSV-2^esi^ SCs duplicated levels of Mock microglia. These results suggested that, despite the immune infiltration, C9orf72 protein levels were decreased in HSV-2^esi^ SCs and microglial lysosomes were altered in a similar way to C9orf72 deficient mice and the SC of C9ORF72 ALS patients.

## Discussion

Genetic alteration of some key genes like *APP*, *MAPT*, or *HTT* are sufficient to cause a NDDs. However, the majority of NDDs cases are sporadic, and the most probable cause is a combination of genetic and environmental factors [2]. Genetic risk factors identified by GWAS have demonstrated the involvement of the IS in NDDs [55], and the innate immunity has become a central player for NDDs [54]. Thereby, any environmental factor capable of altering the IS within the CNS must be studied as a potential risk factor for NDDs. Neurotropic viruses, especially HSV, have been proposed for a long time to be risk factors for NDDs [3]. However, this association has never been proved and it is a matter of great controversy, although recent studies are shedding light on this question [14–18].

Different NDDs affect different types of neurons and different areas of the CNS [2]. If HSV are really a potential risk factor for NDDs the site of infection will be a crucial factor, as the innervation of the infection site will determine which area of the CNS will be accessed by HSV, leading to different potential NDD scenarios. Here, we have used HSV-2 vaginal infections as a natural route to get access into the SC, with the final goal to determine whether HSV-2 has a role in ALS, a disease affecting motor neurons of the SC.

HSV-2 latent infection after vaginal infection caused a severe infiltration of leukocytes in the SC in laminae I and II within lumbar 6, sacral 1, and sacral 2 regions. Neurons from laminae I and II receive input from Aδ and C sensory neurons [60, 61], suggesting that HSV-2 reached the SC through the lumbo-sacral dorsal root ganglia. We also observed frequent CD45^high^ cells infiltrated in the intermediate gray in sacral 2 region, suggesting that HSV-2 may also reach the SC through the parasympathetic innervation of genital organs [52]. Finally, and importantly for the goal of this study, we observed the presence of CD45^high^ in the lamina IX, where motor neurons are located. We hypothesize that CD45^high^ cells arrive to lamina IX because HSV-2 is capable of infect motor neurons, either through limb skeletal muscles after a deep genital infection or through infected sensory neurons and spinal cord circuits [62]. This presence of CD45^high^ cells in close proximity to motor neurons remained to the immune infiltration in the SC of ALS patients [27, 31]

In addition to leukocyte infiltration, we have observed the presence of endogenous antibodies in the SC during HSV-2 latency, in agreement with previous reports [63]. Endogenous antibodies reaching the CNS, together with the infiltration of mouse FcR^+^ cells during HSV-2 latent infection, compromised the use of antibodies produced in mice as cellular and molecular markers, even using specific blockage techniques (not shown). For this reason, we decided not to use mouse antibodies for this study, a decision that limited our access to molecular markers but increased the reliability and reproducibility of our results.

Glial cells were also altered in HSV2^esi^ SCs. We observed moderate changes in astrocytes in infected SCs, with increased levels of GFAP and signs of astrocytosis seen by IFs (not shown). However, major changes occurred in microglia. In latently infected SCs, microglia were increased in number and altered in morphology across the entire lumbo-sacral region. At the cellular level, microglia showed increased lysosomal area, and at the molecular level, some microglial cells expressed high levels of MHC-II. We did not find markers of pro-inflammatory or anti-inflammatory activation in microglia, but we found solid data suggesting that microglia during HSV-2 latency is different from homeostatic microglia. It is plausible that microglia in infected SCs have been primed [64] during productive infection, after being exposed to IFNs and other cytokines. Priming of microglia would prepare microglia against HSV-2 reactivations. Regarding that, “MHC-II hot-spots” would be HSV-2 attempts of reactivation in infected neurons detected by microglia. These attempts are not unusual in HSV mouse models [11, 12], although we might expect these “MHC-II hot-spots” may occur more frequently in human CNS, as humans are the natural host for HSV-2. Importantly, we observed “MHC-II hot-spots” close to motor neurons and SC microgliosis with high expression of MHC-II was one of the earliest observation of neuroimmunological changes in ALS [27] and loss of the homeostatic microglial state is now seen as a major early event in NDDs [64–67].

Changes in microglia may also explain the decrease of C9orf72 protein levels in infected SCs. C9orf72 is necessary for the correct function of homeostatic microglia, and its total deficiency promotes microglial over-activation among other immune dysregulations [31, 43–46]. Then, we could hypothesize that low levels of C9orf72 may be required to maintain some degree of microglial activation to control HSV-2 latent infection. The Interferome Database shows that C9ORF72 mRNA is decreased in human MΦ upon IFNγ stimulation, suggesting an antiviral function of low levels of C9ORF72 [68]. More importantly, it has been shown that C9ORF72 suppresses STING-induced inflammation in myeloid cells [46], and C9ORF72 deficiency leads to a marked early activation of the type I interferon response [46]. In line with this hypothesis, a recent report suggests that disruption of the lysosome regulatory complex Smcr8-Wdr41-C9orf72 increases the sensitivity of endosomal Toll-Light-Receptors (TLRs) [69]. Then, by lowering levels of C9orf72, microglia would be able to increase the sensitivity of TLR3 and TLR9 and early type I interferon response, all critical elements for HSV detection [51, 70, 71], and in this manner, be prepared for potential HSV reactivations. However, it is unknown, the long-term consequences of a decrease of C9orf72 in the SC.

During ALS, motor neuron in the SC degenerate and die [24, 25]. In infected SCs, we have not observed changes in the number of motor neurons. In fact, no changes in neurons were found in the SC. ALS major histopathological hallmark is the hyperphosphorylation and cytoplasmic accumulation of the nuclear RBP TDP-43. We have found an increase in protein levels of TDP-43 by WB in infected SCs. By IF, we could observe that TDP43 remained nuclear and unaltered in all neurons analyzed. Instead, we found many CD45^high^ being positive for TDP-43 that would explain the result obtained by WBs. A similar situation occurred with the ALS-related protein Fus. However, a complementary explanation would be taken in consideration. High amounts of the lariat intron LAT are found in HSV latently infected neurons [10, 13]. TDP-43 and Fus have special affinity for long intronic RNA sequences [72]. Then, it is plausible that HSV-2 latently infected neurons may express higher levels of TDP-43 and Fus in response to the presence of LAT. In line with this hypothesis, bioinformatics predict several binding sites for TDP-43 and Fus in the LAT sequence (not shown), and curiously, a recent report suggests antiviral responses may trigger Fus aggregation [73].

Our model of latent HSV-2 infection of the SC has shown neuroinflammatory features. This neuroinflammation, at the early latency, was resembled to the immune alteration observed in the SC of the ALS patients, such as leukocyte infiltration and microglia alteration with high levels of MHC II in close proximity to motor neurons. Also, it is important to note that the lysosome alteration in microglia observed in HSV-2 infected SC resembled to the lysosome alteration in microglia described in C9ORF72 ALS patients. However, no neuropathological ALS hallmarks were found in motor neurons in HSV-2 infected SCs. This strongly suggests that HSV-2 latent infection is not sufficient to cause ALS, although neuroinflammatory changes observed in the SC raises the possibility that HSV-2 may be a risk factor for ALS. Further experiments combining our model with aging and/or different ALS genetic mouse models, may solve this question.

## Conclusions

To study whether HSV-2 may be related to ALS, we have set up a mouse model to study long-term effects of HSV-2 latent infection and its associated neuroinflammation in the SC. In this model, we have observed a leukocyte infiltration, an alteration of the microglia, and decreased levels of C9orf72 in the SC. Inflammation caused by latent infection of HSV showed some similarities to inflammation observed in the SC of ALS patient, although no changes mimicking ALS neuropathology, like TDP-43 or Fus cytoplasmic inclusions in motor neurons, were found. Then, further studies need to be performed to test whether HSV-2 has a role in ALS.

## Data Availability

Not applicable.
